# The perfused swine uterus model: long-term perfusion

**DOI:** 10.1186/1477-7827-10-110

**Published:** 2012-12-15

**Authors:** Klaudija Geisler, Julian Künzel, Philipp Grundtner, Andreas Müller, Matthias W Beckmann, Ralf Dittrich

**Affiliations:** 1 Department of Gynecology, Erlangen University Hospital, Erlangen, Germany

**Keywords:** Swine uterus, Perfusion, Long-term, Survival, Perfusion model

## Abstract

**Background:**

It has previously been shown that the viability of swine uteri can be maintained within the physiological range in an open perfusion model for up to 8 hours. The aim of this study was to assess medium- to long-term perfusion of swine uteri using a modified Krebs–Ringer bicarbonate buffer solution (KRBB) in the established open perfusion model.

**Methods:**

In an experimental study at an infertility institute, 30 swine uteri were perfused: group 1: n = 11, KRBB; group 2: n = 8, modified KRBB with drainage of perfusate supernatant; group 3: n = 11, modified KRBB with drainage of perfusate every 2 h and substitution with fresh medium. Modified and conventional KRBB were compared with regard to survival and contraction parameters: intrauterine pressure (IUP), area under the curve (AUC), and frequency of contractions (F).

**Results:**

Modified KRBB showed significantly higher IUP, AUC, and F values than perfusion with conventional KRBB. In group 3, the organ survival time of up to 17 h, with a 98% rate of effective contraction time, differed significantly from group 1 (*P* < 0.001).

**Conclusions:**

Using modified KRBB in combination with perfusate substitution improves the open model for perfusion of swine uteri with regard to survival time and quality of contraction parameters. This model can be used for medium- to long-term perfusion of swine uteri, allowing further metabolic ex vivo studies in a cost-effective way and with little logistic effort.

## Background

Experimental organ perfusion originated in the field of transplantation medicine, where it was used to investigate physiological, pathophysiological, and metabolic processes in tissue and cells [1-3]. Studies of the liver, lungs, and kidney using long-term perfusion have been successfully carried out and reported [2,4,5]. Various perfusion solutions have been used for organ maintenance; Iwasaki et al., for example, used a Krebs–Henseleit–Ringer medium for long-term perfusion of isolated rat hearts [6].

In 1986, Bulletti et al. developed an open extracorporeal perfusion system, without recirculation of the medium, for the human uterus [7,8]. They carried out various in vitro studies on extirpated uteri, such as hormonal analyses in the endometrium and myometrium and assessment of electromechanical activity in the smooth muscle. They also examined hormonal influences on uterine contractility during different phases of the menstrual cycle [9,10]. The first early human pregnancy in a perfused uterus was achieved in 1987 [8]. On the basis of the developments by Bulletti et al., Dittrich and Maltaris in 2003 established a model for the perfusion of swine uteri [11]. The use of swine uteri in the perfusion model is suitable for investigating the effects of a wide variety of medications in large numbers of organs in physiological conditions. The results are well comparable with human conditions. Although there are anatomic and physiological differences between the human and swine uterus, the microscopic structures of the organs are similar. The regulatory control system for endocrine functions also allows direct comparison. It was shown in these studies that vitality parameters could be maintained within the physiological range for up to 8 hours in optimal experimental conditions [11].

Topics investigated using the swine uterus perfusion model have included the influence of oxytocin, by Dittrich et al. [11]; the roles of estrogen, progesterone [12], and prostaglandins, by Müller et al. [13]; and the effect of spasmolytics on contractility in the swine uterus, by Künzel et al. [14]. The model has also been used to analyze the mechanisms of intrauterine transport towards the dominant follicle [15]. Richter et al. [16] published the results for long-term perfusion using a closed perfusion model of the human uterus and with a modified Krebs–Ringer bicarbonate buffer (KRBB) solution in normothermia. A total of 25 human uteri were studied for up to 24 hours, with preservation of cell integrity and thus in physiological conditions. The use of a closed system, with recirculation of the medium, led to a marked reduction in the amount of perfusate required in comparison with the studies by Bulletti et al. As reported by Dittrich et al., the vitality of swine uteri can be maintained in an open perfusion model for up to 8 h [11].

The aim of the present study was to investigate medium-term to long-term perfusion of swine uteri using the established open perfusion model. In the future, it is intended to use the swine uterus in vitro to investigate more detailed physiological and metabolic issues, e.g. the role of M_3_- cholinergic receptors. For this purpose, an experimental approach was used to compare a modified KRBB solution as described by Richter et al. [16] with conventional KRBB with regard to the survival period achievable in swine uteri.

## Methods

### Swine uterus

Swine uteri were collected from the abattoir in Erlangen in the early morning, immediately after killing (with electric shocks, 1.5 A, 400 V, 4 s). The organs were all from healthy swine aged 5–18 months. One uterus per day was selected for the experiments on the basis of size, weight, general condition, and cannulability of the uterine arteries. Any experimental research that is reported in the manuscript has been performed with the approval of the ethics committee of the University of Erlangen- Nuremberg.

### Cannulation

**Figure 1:**
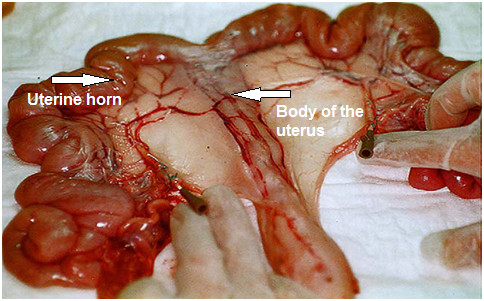
A bilaterally cannulated swine uterus (reproduced with permission from ref. 17).

The selected uterus was dissected in a standardized way for cannulation. The uterine arteries were released from the surrounding connective tissue in the broad ligament of the uterus bilaterally as far as the adventitia. This was followed by cannulation of the arteries using 16-G Abbocaths, which were fixed in place using Vicryl 3–0 sutures (Ethicon Johnson & Johnson International Inc., Brussels) (Figure 1). Correct positioning of the Abbocaths and patency of the uterine vascular system were checked using careful rinsing of the two arteries, each with 2.5 mL NaCl 0.9%. An intra-arterial injection of a heparin solution (2500 IU in 5 mL NaCl 0.9%; Braun Heparin Sodium 25000 IU vial; B. Braun Melsungen Ltd., Melsungen, Germany) was carried out to prevent thrombus formation and to rinse out any already coagulated blood. In addition, the tubes and the vascular plexus surrounding the ovary were ligated to prevent the perfusion solution from escaping at these points and to establish and maintain better uterine perfusion pressure. This was also intended to minimize any disturbing hormonal influences by estrogens and gestagens from the ovaries.

### Perfusion system

**Figure 2:**
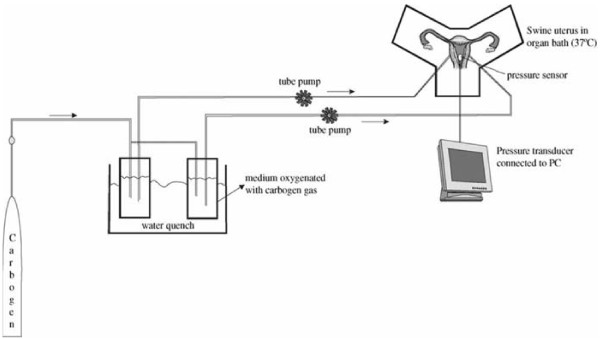
Diagram showing the set-up of the open perfusion system (reproduced with permission from ref. 17).

Following cannulation, the organ that had been prepared for perfusion was placed in an organ bath at a set temperature. The organ bath initially consisted of 1 L of perfusion medium warmed to 37°C. During the entire experiment, the temperature was kept constant at between 36.5°C and 37.5°C (temperature measurement probe, Raumedic; Rehau Ltd., Rehau, Germany). The perfusion medium was warmed to a constant 37°C in a shaking water bath and oxygenated with carbogen (95% O_2_, 5% CO_2_; Linde Inc., Frankfurt, Germany). The Abbocaths were connected with a silicon tube system. A roller pump (Heidolph Ltd., Kelheim, Germany) transported the medium in the tube system at a constant flow rate into the organ’s arterial system. The flow rate was increased gradually over 10–15 min to approximately 15 mL/min. The perfusate flowed continuously through the uterus’s open vascular system into the organ bath — i.e., there was no recirculation of the medium into the arterial system. The diagram in Figure 2 shows the experimental design.

### Perfusion medium

#### Krebs–Ringer bicarbonate buffer solution

The physiological KRBB has been successfully used by Dittrich et al. and by Müller et al. since 2003 for experiments in the perfused swine uterus [11]. The solution is prepared in the institution in Erlangen in accordance with instructions by the manufacturers Sigma-Aldrich Ltd. (Steinheim, Germany) (Table 1).

**Table 1:** Quantities of substances required for single-concentration solutions of the perfusion media used

**Krebs–Ringer bicarbonate buffer solution**		**Modified Krebs–Ringer bicarbonate buffer solution**	
NaCl	7.00 g/L	NaCl	6.896 g/L
KCl	0.34 g/L	KCl	0.372 g/L
MgCl_2_	0.05 g/L	MgSO_4_ + H_2_O	0.246 g/L
CaCl + 2H_2_O	0.05 g/L	CaCl_2_ + 6H_2_O	0.547 g/L
Na_2_HPO_4_	0.10 g/L	KH_2_PO_4_	0.136 g/L
NaHCO_3_	1.26 g/L	NaHCO_3_	2.305 g/L
Glucose + H_2_O	1.98 g/L	d-Glucose	1.500 g/L
NaH_2_PO_4_ + H_2_O	0.18 g/L	Saccharose	0.700 g/L
		Glutathione	0.050 g/L
		1,4-Dithiothreitol	0.100 g/L
		Regular insulin	50 IU/L

#### Modified Krebs–Ringer bicarbonate buffer solution

This perfusion solution, a modified form of KRBB, was previously used by Richter et al. for extracorporeal medium-term to long-term perfusion of human uteri with recirculation of the perfusate [16]. The vitality and functionality of the tissue was maintained for a period of up to 24 h with this solution [16-18]. The composition of the modified solution (Table 1) ensured an isoosmolar ion concentration and an almost physiological pH value (7.36–7.44) and maintained a colloid osmotic pressure of approximately 24 mmHg in the vessels. Saccharose, as an impermeable sugar, was used to prevent edema formation in the tissue. The tripeptide glutathione served as a reducing agent and radical scavenger [19]. Equilibrium between its reduced and oxidized forms was maintained using dithiothreitol. Regular insulin (Actrapid® HM NovoLet® 3 mL 100 IU/mL injection solution; Novo Nordisk Pharma, Ltd.) was also added to the perfusion medium to support metabolization of glucose. In contrast to the method described by Richter et al. [16], gentamicin (Refobacin) was not used. The agents were dissolved in 1 L of distilled water and well mixed in a shaking bath at 37°C.

### Intrauterine pressure measurement

**Figure 3:**
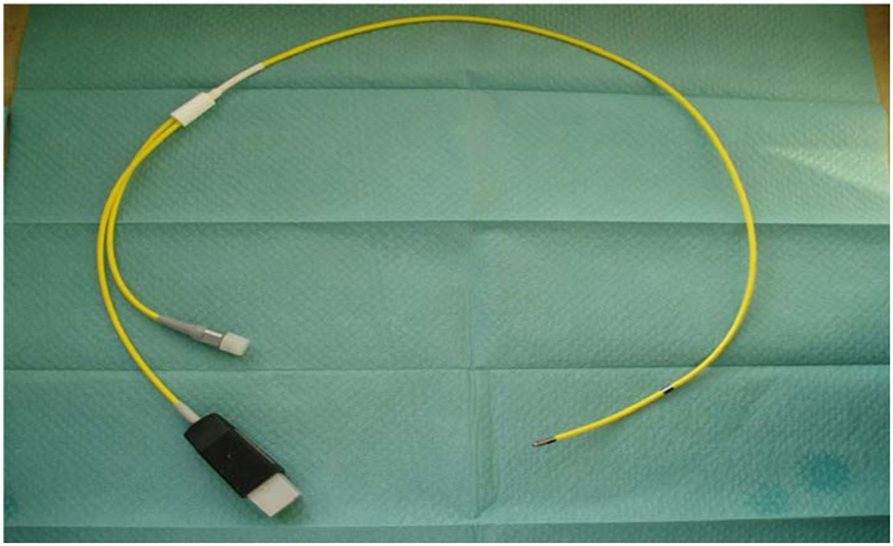
A double-chip microcatheter (Urobar 8 DS-F, Raumedic; Rehau & Co. Ltd., Rehau, Germany).

Intrauterine pressure (IUP) was recorded using a double-chip microcatheter (Urobar 8 DS-F; Raumedic, Rehau Ltd., Rehau, Germany) (Figure 3). The measurement sensors were incorporated into the catheter at intervals of 8 cm, so that after careful introduction, the distal sensor (IUP1) was positioned without tension in the one uterine horn and the proximal sensor in the body of the uterus (IUP2) (Figure 1). The pressure catheter was then fixed in place with a single button suture at approximately 11 cm to prevent it from sliding out during the measurements. After this, the pressure catheter was connected with the monitoring device (Datalogger, MPR1; Raumedic, Rehau Ltd., Rehau, Germany) using a connecting cable.

The data logger allowed simultaneous recording of pressure changes at both measurement points (IUP1, IUP2) and continuous temperature measurement. A pressure value in millimeters of mercury was recorded by the Datalogger for each second. Pressure conditions in the vascular and tube system were displayed using a central venous pressure measurement module.

The measurement electrodes IUP1 and IUP2 and central venous pressure were calibrated to 0 mmHg at the start of each experiment and the corresponding recording. The course of the experiment was observed on a graphic display and finally evaluated with the help of the corresponding software (Datalogg; Raumedic, Rehau Ltd., Rehau, Germany).

### Data processing and statistics

#### Data processing

For each uterus, data for time intervals of 10 min, 20 min after the start of the experiment, after 1 hour and every hour up to the end of the experiment (objective death of the uterus) were analyzed. Additional evaluation of the data was carried out using the Origin program (OriginLab version 8.5; OriginLab Corporation, Northampton, Massachusetts, USA).

Absolute maximum pressures (IUP) and areas under the curve (AUCs) were calculated. The amount of the AUC, as the total of all pressure values measured over a specific time interval, represented the work performed and output of the myometrium. In addition, the relative or effective contraction time (= contraction time/perfusion time) was calculated. The number of contractions per time interval was evaluated as the contraction frequency (F). The time from beginning of measurable contractions to the point of absence of measurable uterine contractions or absence of rhythmic contractions was calculated as survival time in hours.

#### Statistics

The data calculated by the Origin program were further analyzed statistically using Microsoft Excel and SPSS (IBM SPSS Statistics, version 19.0.0.1). Group comparisons were carried out after checking for normal distribution, the prerequisites applying in each case, the scale level of each of the attributes analyzed and the corresponding test situation, using the Mann–Whitney *U* test and the Kruskal–Wallis test or one-way analysis of variance (significance level *P* = 0.05; post-hoc *P* = 0.017). In the post-hoc procedure, the Tukey and Tamhane test was applied, depending on the results of tests for variance homogeneity and the test procedures previously used.

### Experimental procedure

The uteri were divided into three groups for the experiment (Table 1):

● In group 1, 11 uteri were perfused with conventional KRBB.

● In group 2, eight uteri were perfused with the modified KRBB. In addition, excess perfusate was drained in the basin in this group.

● In group 3, 11 uteri were also perfused with the modified KRBB. In addition, the collected perfusate was drained from the organ basin approximately every 2 h physically, using gravity, and replaced with fresh medium.

#### Induction of rhythmic uterine contractions

To achieve optimal saturation of the uterus with oxygen, nutrients, and electrolytes, the initial perfusion was maintained for approximately 1 hour. If no spontaneous uterine contractions were recorded during this phase, oxytocin (Syntocinon® 10 IU = 50 mg/mL; Novartis Pharma, Nuremberg, Germany) was administered as a bolus through the tube system at increasing dosages of 0.1 IU, 0.5 IU, and 1 IU at intervals of 15 min each until rhythmic contractions were induced [11]. If rhythmic contractions were still not observed, the experiment was stopped.

#### Vitality parameters

At the start of the experimental series and at 1-hour intervals, measurements of vitality parameters were made in all of the organs using the blood gas analysis device (ABL 800 Flex; Radiometer Ltd., Willich, Germany). pH and lactate were measured from venous drainage fluid.

#### End of the experiment

The end point of each experiment was defined as the absence of measurable uterine contractions or absence of rhythmic contractions after administration of oxytocin boluses at increasing dosages of 0.1 IU, 0.5 IU, and 1 IU at intervals of 15 min each.

## Results

A total of 30 uteri were perfused. The mean weight of the organs was 152 g (range 75–200 g, median 160 g).

### Group 1: KRBB

**Figure 4:**
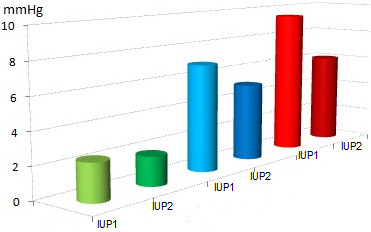
**Mean values (mmHg) for IUP1 and IUP2.** Group 1: green; 2.37 mmHg at IUP1 and 1.86 mmHg at IUP2, group 2: blue; 6.9 mmHg at IUP1 and 5.1 mmHg at IUP2, group 3: red; 9.45 mmHg at IUP1 and 6.0 mmHg at IUP2.

**Figure 5:**
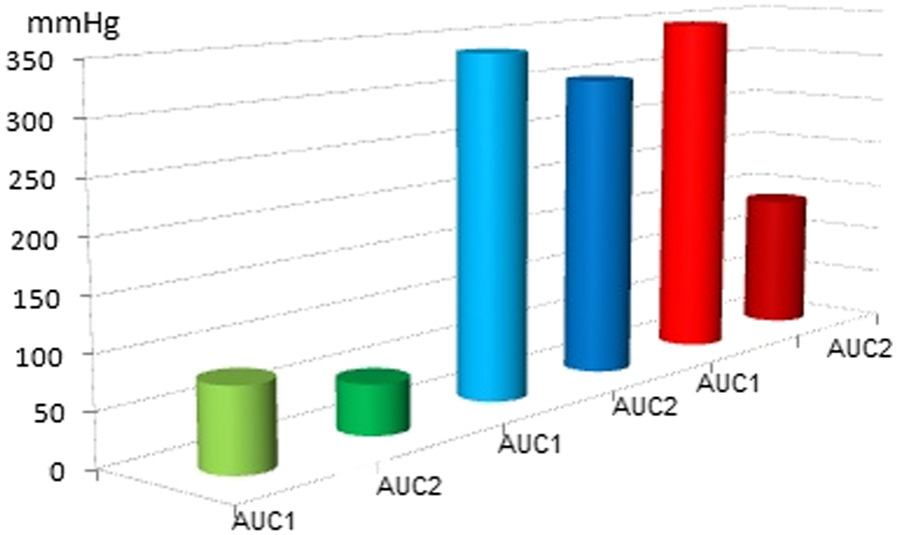
**Mean values (mmHg) for AUC1 and AUC2.** Group 1: green; AUC1 was 78.15 mmHg and AUC2 was 48.11 mmHg, group 2: blue; AUC1 was 335.3 mmHg and AUC2 was 297.4 mmHg, group 3: red; AUC1 was 345.2 mmHg and AUC2 was 137 mmHg.

**Figure 6:**
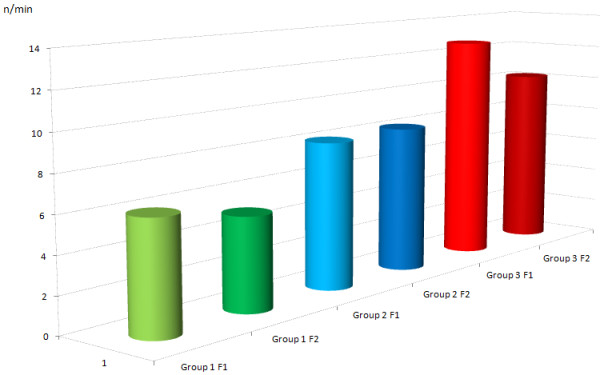
**Mean values (n/time interval) for contraction frequency (F).** Group 1: green; F1 was 6.0 and F2 was 5.1, group 2: blue; F1 was 8.13 and F2 was 8.19, group 3: red; F1 was 12.7 and F2 was 10.2.

**Figure 7:**
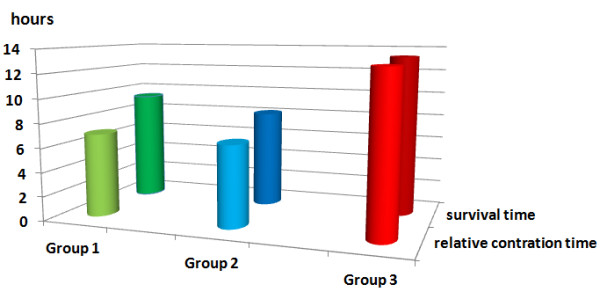
**Mean survival time (back row) and mean effective contraction time (front row).** Group 1: green; survival time was 9.3 h and effective contraction time was 6.95 h, group 2: blue; survival time was 8.13 h and effective contraction time was 6.73 h, group 3: red; survival time was 13.3 h and effective contraction time was 13 h.

**Figure 8:**
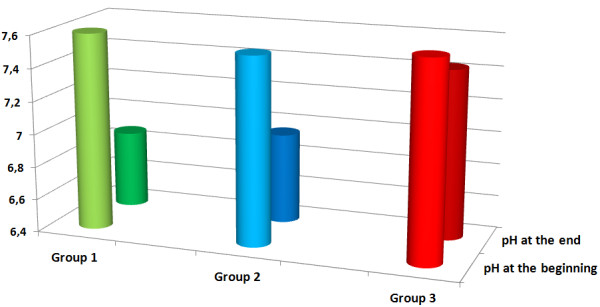
**Mean pH values after 1 h perfusion (front row) and at the end of the experiment (back row).** Group 1: green; pH after 1 hour was 7.6, changing to 6.9 up to the end, group 2: blue; pH after 1 h was 7.54, changing to 7.0 up to the end, group 3: red; pH was 7.6 after 1 hour and it remained constant at 7.4 up to the end.

**Figure 9:**
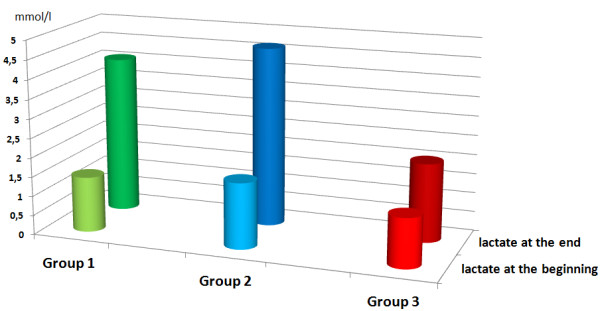
**Mean lactate values (mmol/L) after 1 h perfusion (front row) and at the end of the experiment (back row).** Group 1: green; lactate increased from 1.43 mmol/L to 4.1 mmol/L, group 2: blue; lactate increased from 1.7 mmol/L to 4.6 mmol/L, group 3: red; lactate increased from 1.27 mmol/L to 2.0 mmol/L.

A total of 11 uteri, with a mean initial weight of 168 g (range 130–200 g, median 170 g), were perfused with conventional KRBB (Table 1). Initial rhythmic contractions were induced using oxytocin in all of the organs. The mean pressures were 2.37 mmHg at IUP1 and 1.86 mmHg at IUP2 (Figure 4). The mean AUC1 and AUC2 were 78.15 mmHg and 48.11 mmHg, respectively (Figure 5). The mean frequency 1 (F1) was 6.0 contractions and the mean frequency 2 (F2) was 5.1 contractions per time interval (Figure 6). Macroscopically visible contractions were seen on average after a latency period of 2.35 h. The mean survival time was 9.3 h (range 7–11 h, median 9 h). The organs’ effective contraction time showed a mean of 6.95 h — i.e., contractions occurred during 75% of the perfusion time (Figure 7). The mean pH value after 1 hour of perfusion was 7.6, changing to 6.9 up to the end of the perfusion period (Figure 8). The mean lactate value correspondingly increased from 1.43 mmol/L to 4.1 mmol/L (Figure 9).

### Group 2: modified KRBB

A total of eight uteri, with a mean initial weight of 147 g (range 70–200 g, median 160 g), were perfused with the modified KRBB. Initial contractions were induced in two organs using oxytocin. The mean pressures were 6.9 mmHg at IUP1 and 5.1 mmHg at IUP2 (Figure 4). The mean AUC1 was 335.3 mmHg and the mean AUC2 was 297.4 mmHg (Figure 5). The mean F1 and F2 were 8.13 and 8.19 contractions per time interval, respectively (Figure 6). Macroscopically visible contractions started on average after a latency period of 1.4 hours. The mean survival time was 8.13 hours (range 6–11 h, median 8 h). The organs’ effective contraction time showed a mean of 6.73 h — i.e., contractions took place during 83% of the perfusion period (Figure 7). The mean pH value after 1 h was 7.54, changing to 7.0 up to the end of the perfusion period (Figure 8). The mean lactate value correspondingly increased from 1.7 mmol/L to 4.6 mmol/L (Figure 9).

### Group 3: modified KRBB and substitution of the total perfusate in the organ basin with fresh perfusion medium

A total of 11 uteri, with a mean initial weight of 143.5 g (range 75–200 g, median 145 g), were perfused with the modified KRBB (Table 1) and in addition the perfusate collected in the organ basin was completely replaced with fresh medium approximately every 2 hours. In this group it was not necessary to induce initial contractions by oxytocin. The mean pressure values were 9.45 mmHg at IUP1 and 6.0 mmHg at IUP2 (Figure 4). The mean AUC1 was 345.2 mmHg and the mean AUC2 was 137 mmHg (Figure 5). The mean F1 and F2 were 12.7 and 10.2 contractions per time interval, respectively (Figure 6). Macroscopically visible contractions were visible after a mean latency period of 0.3 hours. The survival time was 13.3 h (range 10–17 h). The organs’ effective contraction time averaged 13 h — i.e., contractions took place during 98% of the perfusion period. The mean pH value was 7.6 after 1 hour and it remained constant at 7.4 up to the end of the perfusion period (Figure 8). The mean lactate value increased from 1.27 mmol/L to 2.0 mmol/L (Figure 9).

### Statistically evaluation of the data

Significantly different values for the contraction parameters IUP, AUC, and F were seen in each case in groups 2 and 3 in comparison with perfusion using conventional KRBB in group 1 (Table 2). Initial rhythmic contractions were already observed after perfusion with the modified KRBB after 1.4 h (group 2) and after 0.3 h (group 3). In both group 1 and group 2, a clear increase in lactate and a decline in the pH value occurred during the course of the experiment. In group 3, regular substitution of the entire drained perfusate with fresh nutrient medium led to maintenance of a constant acid–base balance. This was reflected in significant differences in lactate and pH values in comparison with group 1 (*P* = 0.005 and *P* < 0.001, respectively). The survival time in group 3 of up to 17 hours (mean 13.3 h, median 13.5 h), with a 98% proportion of effective contraction time, was significantly different from group 1 (*P* < 0.001). The *P* values are shown in Table 2 as the results of the group comparisons for the parameters studied and taking each test procedure into account.

**Table 2:** Group comparisons for the parameters studied; significant values are shown in bold type

	**Group 1 vs. Group 2**	**Group 1 vs. Group 3**	**Group 2 vs. Group 3**
IUP1 (mmHg) ^a^	***P*** **= 0.015**	***P*** **< 0.001**	*P* = 0.220
IUP2 (mmHg) ^a^	*P* = 0.073	***P*** **= 0.002**	*P* = 0.397
AUC1 (mmHg/time) ^a^	***P*** **= 0.002**	***P*** **= 0.001**	*P* = 0.703
AUC2 (mmHg/time) ^a^	***P*** **< 0.001**	***P*** **= 0.007**	*P* = 0.379
F1 (contractions/time) ^a^	***P*** **= 0.001**	***P*** **< 0.001**	***P*** **< 0.001**
F2 (contractions/time) ^a^	***P*** **< 0.001**	***P*** **< 0.001**	***P*** **= 0.031**
Lactate start–end (mmol/L) ^b^	*P* = 0.953	***P*** **= 0.005**	*P* = 0.020
pH start–end ^b^	*P* = 0.461	***P*** **< 0.001**	***P*** **= 0.008**
Survival ^c^	*P* = 0.149	*P* < 0.001	*P* = 0.031

^a^ Mann–Whitney *U* test.

^b^ One-way analysis of variance + post-hoc.

^c^ Kruskal–Wallis test.

## Discussion

Adequate uterine contractility and an intact uterotubal transport process are necessary on the one hand for the transport of semen and gametes and also for successful spontaneous or assisted implantation of the embryo. On the other hand, inadequate uterine activity can lead to ectopic pregnancy, a tendency to spontaneous abortion, retrograde bleeding, and endometriosis [20-22]. As mentioned above, our research group has since 2003 therefore been focusing on functional studies of the perfused swine uterus [11]. The open perfusion model makes it possible to measure spontaneous and initiate contractions in the uterine horn and the body of the uterus using a double-chip microcatheter. In a large number of swine uteri, it has been possible to apply agents and medications such as oxytocin, tocolytics, spasmolytics, estrogen, progesterone, prostaglandins and seminal plasma and to examine their effects on the myometrium [11-14,23]. The bicornate swine uterus is particularly suitable for parallel experiments in the same genomic organ — for example, to evaluate transport mechanisms to the side bearing the dominant follicle [15]. The perfusion medium used to date was a standardized KRBB, with which an experimentation period in physiological conditions of up to a maximum of 8 hours was possible (Table 1) [11]. The applicability of perfusion models for experimental studies of placental tissue, ovaries, and uteri has been repeatedly emphasized in the literature [7,24,25].

In the present study, the open perfusion model previously described was to be further developed in order to make it possible in the future to study metabolic receptor ligands such as M3 cholinergic receptors in long-term perfusion of the ex vivo swine uterus. Methods of conserving the organ with hypothermia have severe limitations in connection with metabolic studies, since among other things they lead to inactivation of transmembrane ion pumps [16]. In experimental and clinical transplantation medicine, various solutions have been used for cold conservation of organs, such as Euro-Collins solution, University of Wisconsin solution, histidine-tryptophane-ketoglutarate solution, and HypoThermosol [19,26-30]. Successful use of KRBB for perfusion of human and porcine uteri has been described in many previous literature reports [8,11,16]. As in the studies by Bulletti et al. [8] and Richter et al. [16], a modified KRBB was used for experimental long-term perfusion of swine uteri in the present study.

In comparison with the perfusion system used by Richter et al. [16], with recirculation of the nutrient medium, the open variant of the perfusion system is more cost-effective. It is not necessary to use a membrane oxygenator and an incubator for the experiment. The perfusion medium is continuously oxygenated and the swine uterus is surrounded by nutrient medium in the organ basin. The open perfusion system, consisting of an organ basin, roller pump with infusion system, gas bottle, and shaking bath, as well as software and hardware, can be quickly and effortlessly set up and operated by one person. The use of swine uteri for perfusion experiments is appropriate, as discussed in the introduction, and the results are comparable with human conditions. The logistic effort involved in obtaining the swine uteri is much lower in comparison with perfusion of human uteri, allowing a larger number of organs to be studied in a shorter time. When the perfusate is recirculated, as described by Richter et al. [16], perfusion medium can be saved. With this method, it is nevertheless still necessary to completely replace approximately 2 L of perfusion medium after 4 hours [18]. In the open perfusion model described here, approximately 1.5 L/h is consumed with a flow rate of around 15 mL/min and complete substitution of the basin perfusate with 1 L of fresh medium every 2 h. This corresponds to a perfusion medium consumption of approximately 6 L every 4 h, around three times the amount in comparison with the study by Richter et al. [16]. It should be taken into account here that direct comparison of perfusion medium consumption between human and swine uteri is only possible to a limited extent, due to the different size of the organs. Although the greater consumption of perfusion medium is a disadvantage of the open model, it is quite acceptable in view of other cost savings. Producing the modified KRBB is inexpensive, uncomplicated, and takes little time. Relevant bacterial infection of the organ during the experiment has been assessed as minimal, and antibiotic prophylaxis was not therefore used.

As mentioned earlier, a maximum perfusion time of 8 h has been achieved to date with the standardized form of KRBB [11]. The results of the present study show that using the modified KRBB can significantly prolong the survival time of swine uteri from a maximum of 11 h (in group 1) to a maximum of 17 h (group 3). Against the background of the results reported by Bulletti et al. [8] and Richter et al. [16] with regard to long-term perfusion of human uteri, the aim of maintaining the vitality of swine uteri for 24 h was not achieved. Nevertheless, a mean survival time of 13.3 h in group 3, with a maximum survival time of up to 17 h, now appears capable of permitting more detailed metabolic and physiological studies of the swine uterus. The physiology of the swine uterus is very similar to that of the human uterus, although not identical, so that only limited direct comparison of the survival times reported in the literature is possible. The results show that a combination of modified KRBB and substitution of the entire perfusate in the organ basin appears to be necessary in order to achieve a significant increase in the survival time. In the present study the vitality parameters, pH and lactate, were measured from venous drainage fluid, which is an accurate method to monitor the physiologic condition of the organ [11]. But to investigate the impact of long term perfusion on a cellular level it would be necessary to take biopsies at the beginning and at the end of the experiment.

Significantly better contraction parameters (IUP, AUC, F) were already observed using perfusion with modified KRBB in group 2 in comparison with conventional KRBB in group 1 (Table 2). In addition, the modified KRBB in comparison with conventional KRBB usually led to the initiation of spontaneous contractions after a short latency period and with a much longer effective contraction period (Figure 7). In practice, this means that on the one hand a marked time saving is possible due to the shorter latency period (2.35 h in group 1 vs. 0.3 h in group 3) before the start of spontaneous contraction of the organs, while on the other hand the quality of the perfusion experiments is also improved.

## Conclusions

In summary, the use of the modified KRBB in combination with substitution of the basin perfusate led to a marked improvement in survival time and in the quality of the contraction parameters in swine uteri in the open perfusion model. In the future, this model can be used for medium-term to long-term perfusion in more detailed metabolic perfusion experiments, with little logistic effort and at low cost.

## Abbreviations

AUC: Area under the curve; F: Frequency (of contractions); IUP: Intrauterine pressure; KRBB: Krebs–Ringer bicarbonate buffer (solution).

## Competing interests

The authors declare that they have no competing interests.

## Authors’ contributions

KG and RD have made substantial contributions to conception and design and were responsible for acquisition of data. PG and KG were responsible for analysis and interpretation of data. JK, MWB and AM have been revising the manuscript critically for important intellectual content. MWB and RD has given final approval of the version to be published. MWB was supervisor of the present study. All authors read and approved the final manuscript.

## References

[B1] Toledo-Pereyra LH Pulsatile perfusion is still indicated for kidney preservation Transplantation 1982 34 110 10.1097/00007890-198208000-00013 7135465

[B2] Kamada N Calne RY Wight DG Lines JG Orthotopic rat liver transplantation after long-term preservation by continuous perfusion with fluorocarbon emulsion Transplantation 1980 30 43 48 10.1097/00007890-198007000-00009 6994284

[B3] van der Wijk J Slooff MJ Rijkmans BG Kootstra G Successful 96- and 144-hour experimental kidney preservation: a combination of standard machine preservation and newly developed normothermic ex vivo perfusion Cryobiology 1980 17 473 477 10.1016/0011-2240(80)90057-7 7002468

[B4] Baldan N Toffano M Cadrobbi R Codello L Calabrese F Bacelle L Rigotti P Kidney preservation in pigs using Celsior, a new organ preservation solution Transplant Proc 1997 29 3539 3540 10.1016/S0041-1345(97)01013-0 9414827

[B5] Ohura H Kondo T Handa M Saito R Matsumura Y Okada Y Shimada K Hirose M Horikoshi A Sugita M Functional and histopathologic studies of primate pulmonary allografts preserved for 24 hours with a form of modified extracellular solution J Heart Lung Transplant 1995 14 493 504 7544618

[B6] Iwasaki S Araki H Miyauchi Y Nishi K 24-hour preservation of isolated rat hearts perfused with pyridoxalated hemoglobin polyoxyethylene conjugate (PHP) solution at low temperature Artif Organs 1991 15 78 85 2036066 10.1111/j.1525-1594.1991.tb00764.x

[B7] Bulletti C Jasonni VM Lubicz S Flamigni C Gurpide E Extracorporeal perfusion of the human uterus Am J Obstet Gynecol 1986 154 683 688 3953718 10.1016/0002-9378(86)90630-7

[B8] Bulletti C Jasonni VM Tabanelli S Gianaroli L Ciotti PM Ferraretti AP Flamigni C Early human pregnancy in vitro utilizing an artificially perfused uterus Fertil Steril 1988 49 991 996 3371494 10.1016/s0015-0282(16)59949-x

[B9] Bulletti C de Ziegler D Polli V Diotallevi L Del Ferro E Flamigni C Uterine contractility during the menstrual cycle Hum Reprod 2000 15 Suppl 1 81 89 10.1093/humrep/15.suppl_1.81 10928421

[B10] Bulletti C Jasonni VM Martinelli G Govoni E Tabanelli S Ciotti PM Flamigni C A 48-hour preservation of an isolated human uterus: endometrial responses to sex steroids Fertil Steril 1987 47 122 129 3792566 10.1016/s0015-0282(16)49947-4

[B11] Dittrich R Maltaris T Müller A Dragonas C Scalera F Beckmann MW The extracorporeal perfusion of swine uterus as an experimental model: the effect of oxytocic drugs Horm Metab Res 2003 35 517 522 14517766 10.1055/s-2003-42651

[B12] Mueller A Siemer J Schreiner S Koesztner H Hoffmann I Binder H Beckmann MW Dittrich R Role of estrogen and progesterone in the regulation of uterine peristalsis: results from perfused non-pregnant swine uteri Hum Reprod 2006 21 1863 1868 10.1093/humrep/del056 16517557

[B13] Mueller A Maltaris T Siemer J Binder H Hoffmann I Beckmann MW Dittrich R Uterine contractility in response to different prostaglandins: results from extracorporeally perfused non-pregnant swine uteri Hum Reprod 2006 21 2000 2005 10.1093/humrep/del118 16638817

[B14] Künzel J Geisler K Hoffmann I Müller A Beckmann MW Dittrich R Myometrial response to neurotropic and musculotropic spasmolytic drugs in an extracorporeal perfusion model of swine uteri Reprod Biomed Online 2011 23 132 140 10.1016/j.rbmo.2011.03.026 21757131

[B15] Mueller A Siemer J Renner S Hoffmann I Maltaris T Binder H Beckmann MW Dittrich R Perfused non-pregnant swine uteri: a model for evaluating transport mechanisms to the side bearing the dominant follicle in humans J Reprod Dev 2006 52 617 624 10.1262/jrd.18021 16819258

[B16] Richter O Wardelmann E Dombrowski F Schneider C Kiel R Wilhelm K Schmolling J Kupka M van der Ven H Krebs D Extracorporeal perfusion of the human uterus as an experimental model in gynaecology and reproductive medicine Hum Reprod 2000 15 1235 1240 10.1093/humrep/15.6.1235 10831547

[B17] Maltaris T Dragonas C Hoffmann I Mueller A Schild RL Schmidt W Beckmann MW Dittrich R The extracorporeal perfusion of the swine uterus as an experimental model: the effect of tocolytic drugs Eur J Obstet Gynecol Reprod Biol 2006 126 56 62 10.1016/j.ejogrb.2005.07.026 16202502

[B18] Kim SY Extrakorporale Uterusperfusion zur Untersuchung von Uterotonika in der Geburtshilfe und Reproduktionsmedizin [doctoral dissertation] 2006 Bonn: University of Bonn Available at: http://hss.ulb.uni-bonn.de/2006/0687/0687.pdf

[B19] Southard JH Belzer FO Organ preservation Annu Rev Med 1995 46 235 247 10.1146/annurev.med.46.1.235 7598460

[B20] Bulletti C De Ziegler D Polli V Del Ferro E Palini S Flamigni C Characteristics of uterine contractility during menses in women with mild to moderate endometriosis Fertil Steril 2002 77 1156 1161 10.1016/S0015-0282(02)03087-X 12057721

[B21] Bulletti C de Ziegler D Uterine contractility and embryo implantation Curr Opin Obstet Gynecol 2005 17 265 276 Corrected and republished in: Curr Opin Obstet Gynecol 2006, 18:473–484 10.1097/01.gco.0000169104.85128.0e 15870561

[B22] Kissler S Siebzehnruebl E Kohl J Mueller A Hamscho N Gaetje R Ahr A Rody A Kaufmann M Uterine contractility and directed sperm transport assessed by hysterosalpingoscintigraphy (HSSG) and intrauterine pressure (IUP) measurement Acta Obstet Gynecol Scand 2004 83 369 374 15005785 10.1111/j.0001-6349.2004.00412.x

[B23] Dittrich R Henning J Maltaris T Hoffmann I Oppelt PG Cupisti S Beckmann MW Mueller A Kiesewetter F Extracorporeal perfusion of the swine uterus: effect of human seminal plasma Andrologia 2012 44 Suppl 1 543 549 21950778 10.1111/j.1439-0272.2011.01223.x

[B24] Page KR Perfusion of isolated human placenta Proc Nutr Soc 1991 50 345 347 10.1079/PNS19910044 1749801

[B25] Brännström M Flaherty S Methodology and characterization of an in vitro perfusion model for the mouse ovary J Reprod Fertil 1995 105 177 183 10.1530/jrf.0.1050177 8568758

[B26] Bellamy CA Nicely B Mattice BJ Teaster R Comparative analysis of clinical efficacy and cost between University of Wisconsin solution and histidine-tryptophan-ketoglutarate Prog Transplant 2008 18 166 171 quiz 172 18831481 10.1177/152692480801800304

[B27] Hrabalová M Bachleda P Lubuská L Kojecký Z Zadrazil J Krejcí K Al Jabry S Effect of various protective solutions on function after kidney transplantation Biomed Pap Med Fac Univ Palacky Olomouc Czech Repub 2003 147 197 202 10.5507/bp.2003.029 15037904

[B28] Bessems M Doorschodt BM van Vliet AK van Gulik TM Preservation of rat livers by cold storage: a comparison between the University of Wisconsin solution and Hypothermosol Ann Transplant 2004 9 35 37 15478915

[B29] Collins GM Wicomb WN New organ preservation solutions Kidney Int Suppl 1992 38 S197 202 1405375

[B30] Collins GM What solutions are best? Overview of flush solutions Transplant Proc 1997 29 3543 3544 10.1016/S0041-1345(97)01015-4 9414829

